# Molecular characterization of resistance to Rifampicin in an emerging hospital-associated Methicillin-resistant Staphylococcus aureus clone ST228, Spain

**DOI:** 10.1186/1471-2180-10-68

**Published:** 2010-03-04

**Authors:** Virginie Mick, M Angeles Domínguez, Fe Tubau, Josefina Liñares, Miquel Pujol, Rogelio Martín

**Affiliations:** 1 Microbiology Department, Hospital Universitari de Bellvitge, University of Barcelona, IDIBELL, Feixa Llarga s/n 08907 Hospitalet de Llobregat, Barcelona, Spain; 2 CIBERES (CIBER de Enfermedades Respiratorias), ISCIII, Madrid, Spain; 3 Infectious Diseases Department, Hospital Universitari de Bellvitge, University of Barcelona, IDIBELL, Barcelona, Spain

## Abstract

**Background:**

Methicillin-resistant *S. aureus *(MRSA) has been endemic in Hospital Universitari de Bellvitge, Barcelona, since 1990. During the 1990-95 period the Iberian clone (ST-247; SCC*mec*-I) was dominant. Isolates of clonal complex 5 (ST-125; SCC*mec*-IV) gradually replaced the Iberian clone from 1996 to 2003. A new multiresistant MRSA phenotype showing rifampicin resistance emerged in 2004 and rapidly increased from 25% in 2004 to 45% in 2006. The aims of this study were i) the molecular characterisation of rifampicin resistant MRSA isolates, ii) the study of the rifampicin resistance expression by disk diffusion, microdilution and E-test, and iii) the analysis of the *rpoB *gene mutations involved in rifampicin resistance.

**Results:**

A sample of representative 108 rifampicin-resistant MRSA isolates belonged to a single PFGE genotype, ST-228, SCC*mec *type I and *spa *type t041. Of 108 isolates, 104 (96%) had a low-level rifampicin resistance (MICs, 2 to 4 mg/L) and 4 a high-level rifampicin resistance (MICs, 128 - ≥ 256 mg/L). Disk diffusion and E-test methods failed to identify a low-level rifampicin resistance in 20 and 12 isolates, respectively. A low-level rifampicin resistance was associated with amino acid substitution 481His/Asn in the beta-subunit of RNA polymerase. Isolates with a high-level rifampicin resistance carried additional mutations in the *rpoB *gene.

**Conclusions:**

The emergence of MRSA clone ST228-SCC*mec*I, related to the Southern Germany clone, involved a therapeutical challenge for treating serious MRSA infections. Decreased susceptibility to rifampicin in MRSA strains of ST228-SCCmecI was associated with one or two specific mutations in the *rpoB *gene. One fifth of isolates with low-level rifampicin-resistance were missed by the diffusion methods.

## Background

Methicillin resistant *Staphylococcus aureus *(MRSA) is an important pathogen in Spanish hospitals. The percentage of patients infected or colonised by MRSA among patients with nosocomial *S. aureus *has been estimated between 20.2% and 30.5% in nation-wide multicenter studies [1,2].

In the Hospital Universitari de Bellvitge MRSA has been endemic since 1990. The majority of strains isolated during the 1990-95 period belonged to the multiresistant Iberian clone. By multilocus sequence typing (MLST) the Iberian clone showed an allelic profile or sequence type (ST) 247, carrying the staphylococcal cassette chromosome *mec *(SCC*mec*) type I [3]. Isolates of the Iberian clone exhibited resistance against almost all antibiotics available for MRSA therapy including clindamycin, erythromycin, gentamicin, tobramycin, tetracycline, ciprofloxacin and rifampicin. From 1996 to 2003, the Iberian clone was gradually replaced by isolates of Clonal Complex 5 (ST125 and variants; SCC*mec *type IV) related to the Paediatric clone (ST5; SCC*mec *type IV) [4]. Unlike the Iberian clone, these strains showed only consistent resistance to tobramycin and ciprofloxacin combined with variable resistance to clindamycin and/or erythromycin. Similar trends have been observed in other hospitals in Spain and in other countries such as France, Germany, Belgium or Portugal, with involvement of different clonal lineages [5-10]. MRSA isolates resistant to clindamycin, erythromycin, gentamicin, tobramycin, and ciprofloxacin were detected in 2004. These isolates showed reduced susceptibility to rifampicin (RIF-R), according to the Clinical and Laboratory Standards Institute (CLSI) criteria [11]. This new phenotype of multiresistance differed from that of the Iberian clone on the low level RIF-R and on the tetracycline susceptibility. The frequency of the RIF-R MRSA isolates rapidly increased from 2004 to 2006: 25% (59/237) of all MRSA clinical isolates in 2004, 33% (67/206) in 2005, and 45% (116/256) in 2006. The percentage of RIF-R MRSA decreased to 30% (111/378) in 2007 and 25% in 2008 (75/300).

Rifampicin cannot be used as a single agent to treat MRSA infections because of the rapid selection of resistant mutants [12,13]. However, combinations of rifampicin with other anti-staphylococcal agents such as quinolones [14] or fusidic acid [15] could prevent the emergence of rifampicin resistance during therapy [16]. Rifampicin interacts specifically with the RNA polymerase beta-subunit encoded by the gene *rpoB *[12]. Rifampicin resistance in *S. aureus*, as in other bacteria, is associated with mutations in particular regions (cluster I and II) of the gene *rpoB *[13,17].

The objectives of the present study were: i) to characterise a collection of MRSA isolates expressing this new multiresistant pattern, and to determine whether they represented a novel genotype or they were the current representatives of a previously detected clone, ii) to determine the different levels of the rifampicin resistance by disk diffusion, microdilution and E-test, and iii) to analyse mutations in the *rpoB *gene related to rifampicin resistance.

## Methods

### Hospital setting

The Hospital Universitari de Bellvitge in Barcelona, Spain, is a nearly 900-bed tertiary care teaching centre. It is the reference hospital for a geographical area with a population of approximately 1 million inhabitants. The Hospital Universitari de Bellvitge in Barcelona provides medical and surgical care for adult patients with an average of 25,000 admissions per year. It has six intensive care units with a total of 60 beds and an active organ transplant program.

The control of MRSA in our institution is based on the active screening of patients at risk and contact isolation of infected or colonised patients. In spite of this policy, the average rate of total MRSA among *S. aureus *clinical isolates in our hospital was 24% for the 2004-2007 period (minimum 23% in 2007 and maximum 26% in 2006).

The present study has been approved by the Clinical Research Ethics Committee of the Hospital Universitari de Bellvitge.

### Bacterial strains

Identification of *S. aureus *from clinical samples was performed using conventional tests: catalase, latex agglutination (Microgen Staph, Microgen Bioproducts, Camberley, England) and tube coagulase test (Staph-ase, bioMérieux, Marcy l'Étoile, France).

Two hundred and forty-two non-duplicate isolates resistant to clindamycin, erythromycin, gentamicin, tobramycin, ciprofloxacin and resistant to rifampicin (RIF-R) by the disk-diffusion or the microdilution method were recovered in the Microbiology Department of Hospital Universitari de Bellvitge from January 2004 to December 2006. These strains represented 34% of all MRSA isolated between 2004 and 2006, and were isolated from patients admitted to the different surgical, medical and intensive care units in the hospital. One hundred and eight isolates with rifampicin MIC ≥ 2 mg/L were selected for the present study. The selection included the first isolates available each year (33/59, 29/67 and 46/116 isolates from 2004, 2005 and 2006, respectively) from the different hospital wards affected. The origin of the strains was from blood cultures or catheter-related sites (n = 38), wound swabs (n = 28), respiratory samples (n = 24), exudates (n = 12), nasal swabs (n = 4) and sterile fluids (n = 2). Oral informed consent was given by all patients before taking the clinical specimen. The patient acquisition of MRSA infection or colonisation was prospectively assessed. Five strains with the same resistance pattern but fully susceptible to rifampicin (RIF-S) (MIC 0.012 mg/L) were included in this study. This RIF-S pattern represented about 4% of all MRSA isolated between 2004 and 2006.

### Antimicrobial susceptibility testing

Susceptibility testing of primary MRSA isolates is performed routinely by the disk-diffusion method on Mueller-Hinton 2 agar plates (MH2, bioMérieux) to the following antibiotics: penicillin (10 units), oxacillin (1 μg), cefoxitin (30 μg), erythromycin (15 μg), clindamycin (2 μg), gentamycin (10 μg), tobramycin (10 μg), rifampicin (5 μg), tetracycline (30 μg), trimethoprim-sulfamethoxazole (1.25/23.75 μg), chloramphenicol (30 μg), ciprofloxacin (5 μg), vancomycin (30 μg), teicoplanin (30 μg), quinupristin/dalfopristin (15 μg) and linezolid (30 μg). Disks are supplied by BD BBL (Sensi-Disc; Becton, Dickinson and Company, Sparks, MD 21152 USA). MICs of rifampicin were determined in all strains selected for this particular study (108 RIF-R and 5 RIF-S MRSA strains) by microdilution (from 0.06 to 128 mg/L), following the Clinical and Laboratory Standards Institute (CLSI) recommendations [11], and by E-test (AB biodisk, Solna, Sweden). Isolates were interpreted as susceptible or resistant, according to the CLSI criteria [11].

### Detection of rifampicin resistance-associated mutations

An internal sequence of gene *rpoB *of 432 bp (nucleotides 1216 to 1648) was amplified by PCR. This region includes the rifampicin resistance-determining cluster I (nucleotides 1384-1464, amino acid number 462-488) and cluster II (nucleotides 1543-1590, amino acid number 515-530). The amplification was carried out in 5 RIF-S MRSA strains (rifampicin MICs, 0.012 mg/L), and in a selection of 32 RIF-R strains showing different levels of rifampicin resistance: MICs 2 mg/L, 21 strains; MICs 4 mg/L, 7; MICs 128 mg/L, 2; and MICs ≥ 256 mg/L, 2. The oligonucleotide sequences used were rpoBfor (5'-GTC GTT TAC GTT CTG TAG GTG-3') and rpoBrev (5'-TCA ACT TTA CGA TAT GGT GTT TC-3'). Amplification was carried out in a 50 μl volume containing 30 pmol of each primer, 200 μM deoxynucleoside triphosphates (dATP, dCTP, dGTP and dTTP), 3 μl of a template DNA sample and 1 U of AmpliTaq Gold DNA polymerase (Applied Biosystems, Madrid, Spain). Thermal cycling reactions consisted of an initial denaturation (9 min 30 at 94°C) followed by 35 cycles of denaturation (30 s at 94°C), annealing (30 s at 62°C), and extension (1 min at 72°C), with a final extension (10 min at 72°C). The PCR product was purified (QIAquick PCR purification kit, Qiagen, Madrid, Spain) and analysed by DNA sequencing. Cycle sequencing reactions were made up in a final volume of 20 μl with ABI BigDye Terminator v3.0 Ready Reaction Cycle Sequencing kit, following manufacturer's methodology (Applied Biosystems). The nucleotide sequences obtained were compared to the *rpoB *wild type sequence from *S. aureus *subsp. *aureus *(GenBank accession number: X64172) using the clustalw software http://www.ebi.ac.uk/tools/clustalw/index.html.

Rifampicin-susceptible strains used as controls were: ATCC29213 (rifampicin and methicillin susceptible *S. aureus*) and ATCC700698 (rifampicin susceptible MRSA). Two representatives of the Iberian clone were used as rifampicin-resistant MRSA controls: ATCCBAA44 [18,19] and PER88 [3,19].

### Determination of spontaneous mutation frequency for rifampicin resistance

The determination of spontaneous mutation frequency for rifampicin resistance was aimed at identifying whether the presence of a first mutation conferring low level rifampicin resistance facilitated the acquisition of supplementary mutations responsible for increasing rifampicin MICs. The rifampicin mutation frequency was calculated in reference strain ATCC700698 (MIC 0.006 mg/L) and in two RIF-R MRSA strains carrying the low level resistance mutation His481/Asn (rifampicin MICs of 1.5 and 2 mg/L, respectively). Bacterial strains were cultured in a shaking incubator at 37°C in Luria-Bertani (LB) broth (BD Diagnostics, Spain) until the exponential growth phase. Each strain was plated on the selective and non-selective LB agar plates and incubated at 37°C. Rifampicin selecting concentrations were 2 and 20 mg/L for the reference strain, and 20 mg/L for the RIF-R MRSA strains. In these experimental conditions OD_620 _= 0.125 corresponded to 5 × 10^7 ^cfu/ml. The equivalent to 10^7^, 10^8 ^and 10^9 ^cfu were spread on selective plates, and appropriated diluted samples were plated on non-selective plates. After 24 h to 36 h, colonies that grew on selective and non-selective plates were counted and mutation frequencies were calculated. Three independent experiments were performed to ensure reproducibility.

### Molecular typing

#### Pulsed Field Gel Electrophoresis

(PFGE) was performed after *Sma*I restriction of chromosomal DNA according to Chung *et al*. [20]. Pulses run from 5 s to 15 s for 10 h for block 1, and from 15 s to 60 s for 13 h for block 2 [21]. Isolates with PFGE patterns differing in four or less restriction fragments were considered to be subtypes of a single genotype. Isolates with differences in more than four fragments were ascribed to distinct genotypes [22].

#### SCC*mec *typing

Molecular typing based on the amplification of the mobile region *mec *was performed according to previously described procedures [23,24]. Control strains for SCC*mec *typing were: ATCCBAA44 (SCC*mec *type I) [18,19], ATCCBAA-41 (SCC*mec *type II) [19], ATCCBAA-39 (SCC*mec *type III) [19] and HGSA60 (SCC*mec *type IV-A) [24].

#### Multilocus sequence typing

(MLST). Analysis of the seven housekeeping gene sequences was performed according to previously described procedures http://saureus.mlst.net/[25].

#### *spa *typing

The polymorphic region of protein A was studied according to previously described procedures at http://spa.ridom.de/[26]. The interest region was amplified with primers spa-1113f (5'-TAA AGA CGA TCC TTC GGT GAG C-3') and spa-1514r (5'-CAG CAG TAG TGC CGT TTG CTT-3').

## Results

### Rifampicin resistance levels and associated *rpoB *mutations

The majority (n = 104, 96%) of the 108 RIF-R MRSA isolates, showed rifampicin MICs between 2 and 4 mg/L. Two isolates had rifampicin MICs of 128 mg/L and the remaining two had MICs ≥ 256 mg/L. Corresponding E-test and disk diffusion results are shown in table 1. On the basis of these results and following other authors' categorisation [13,17,27] the strains were classified into categories of rifampicin susceptible (MICs, ≤ 0.5 mg/L), low-level rifampicin resistance (MICs, 1 to 4 mg/L), and high-level rifampicin resistance (MICs, ≥ 8 mg/L). Interestingly, 20 strains with rifampicin MICs of 2 mg/L showed inhibition zones between 20 and 23 mm, borderline to the susceptible CLSI breakpoint (inhibition zones ≥ 20 mm). The five RIF-S MRSA isolates, with the same multi-resistance pattern, had rifampicin MICs of 0.012 mg/L and inhibition zones > 30 mm.

**Table 1:** MICs by microdilution and Etest and disk diffusion inhibition zones in 108 rifampicin resistant MRSA isolates.

Microdilution MICs	No. of strains	E-test (range) MICs	Inhibition zones by disk diffusion No. of strains		
			
			≤ 16 mm	17-19 mm	≥ 20 mm
2 mg/L	97	0.75-2 mg/L	15	62	20
4 mg/L	7	3-4 mg/L	7	-	-
128 mg/L	2	> 32 mg/L	2	-	-
≥ 256 mg/L	2	> 32 mg/L	2	-	-

The mutations in the rifampicin resistance-determining region of *rpoB *gene were studied in 32 RIF-R and in 5 RIF-S MRSA strains. Results are shown in table 2. All 32 strains presented the mutational change 481His/Asn, determined by a mutation in cluster I of *rpoB *gene, conferring a low-level rifampicin resistance. The four isolates with MIC≥ 128 mg/L had an additional amino acid substitution: 468Gln/Lys (n = 1), 477Ala/Thr (n = 2) or 527Ile/Leu (n = 1), associated with a high level rifampicin resistance. Mutational changes 468 and 477 were determined by mutations located in cluster I and substitution 527 was determined by a mutation located in cluster II. RIF-S MRSA isolates, had no mutations related to rifampicin resistance. All isolates, including RIF-S isolates, and 3 (ATCC29213, ATCCBAA44, and PER88) out of 4 control strains, presented a silent mutation in amino acid 498 with the substitution Ala(GCG) per Ala(GCT).

**Table 2:** Level of rifampicin resistance and mutations found in the *rpoB *gene of MRSA isolates and control strains

Genotype (ST/SCC*mec*/PFGE)	Rifampicin MICs (mg/L)	Number of isolates	Nucleotide mutation	Amino acid substitution
**ST228/IV-A/A**	**0.012**	5	None	

**ST228/I/B**	**2-4**	28	CAT→AAT	481His→Asn

**ST228/I/B**	**128**	2	CAT→AAT GCT→ACT	481His→Asn 477Ala→Thr

**ST228/I/B**	≥ **256**	1	CAT→AAT CAA→AAA	481His→Asn 468Gln→Lys

**ST228/I/B**	≥ **256**	1	CAT→AAT ATT→CTT	481His→Asn 527Ile→Leu

**ST247/I** **PER88** **(Iberian clone)**	≥ **256**	1	CAT→AAT TCA→TTA	481His→Asn 529Ser→Leu

**ST247/I** **ATCCBAA44** **(Iberian clone)**	**2**	1	CAT→AAT	481His→Asn

### Frequency of spontaneous mutation for rifampicin resistance

The rifampicin mutation frequency was calculated in reference strain ATCC700698 (MIC, 0.006 mg/L) and in two RIF-R MRSA strains carrying the low-level resistant amino acid substitution 481His/Asn (rifampicin MICs, 1.5 and 2 mg/L, respectively). Rifampicin high level resistant mutants occurred with frequencies of around 10^-7 ^to 10^-8 ^in the RIF-R MRSA strains after selection by rifampicin concentration of 20 mg/L. An identical mutational ratio was found in the control strain ATCC700698 at both selective concentrations (2 and 20 mg/L).

### RIF-R MRSA genotypes by PFGE and epidemiology

**Figure 1:**
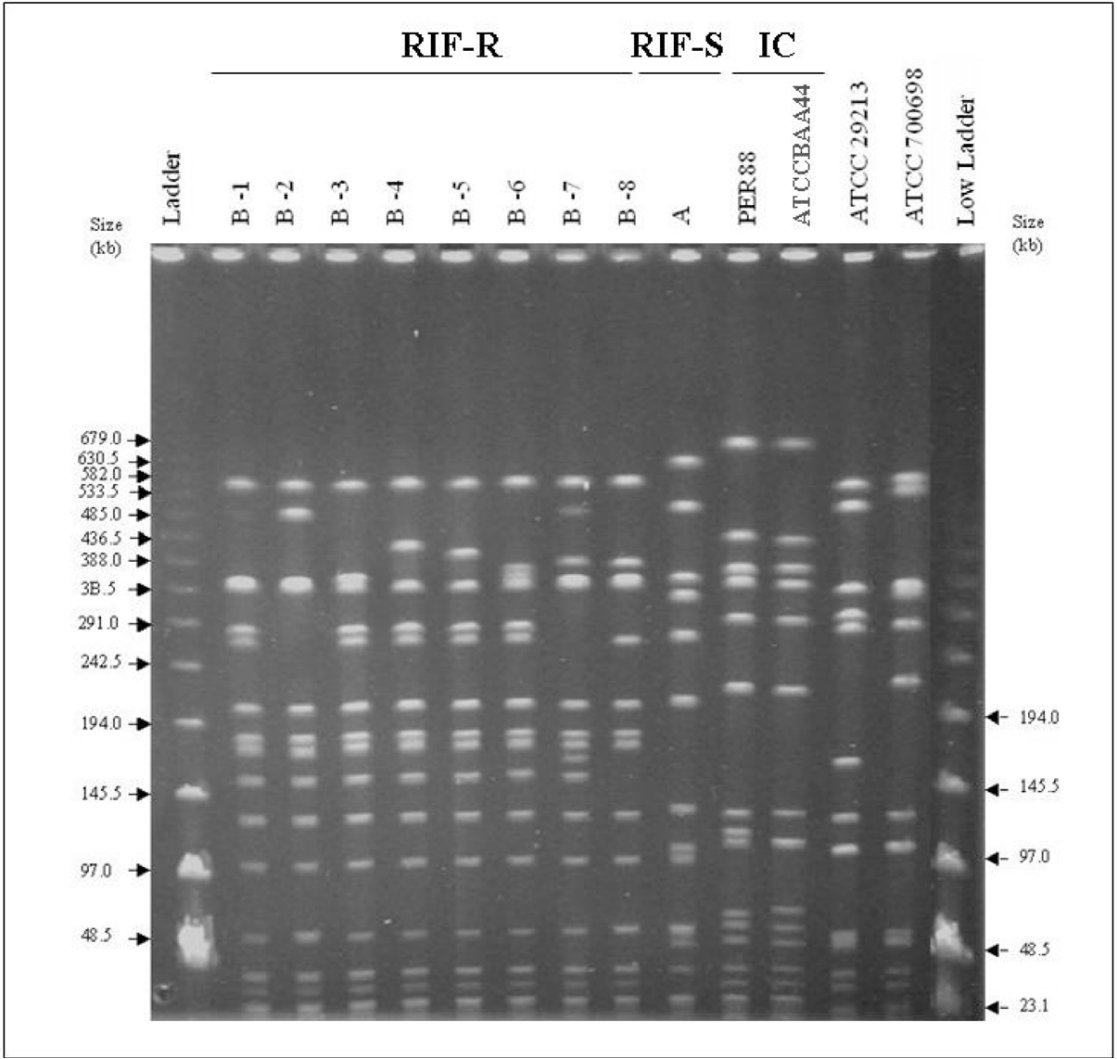
**PFGE subtypes of MRSA strains with decreased susceptibility to rifampicin (RIF-R), "B-1" to "B-8"**. PFGE pattern "A" corresponds to a rifampicin susceptible MRSA isolate (RIF-S). PFGE patterns of controls are shown: Iberian clone (IC) representatives (PER88 and ATCCBAA44), ATCC2913 and ATCC70069.

All 108 RIF-R MRSA isolates belonged to the same genotype by PFGE. This specific restriction pattern (B) was unique, distinct from both the PFGE patterns obtained for the multi-resistant RIF-S MRSA isolates (A) and from representatives of the Iberian clone (figure 1). The RIF-R MRSA isolates were classified into eight subtypes (B-1 to B-8) with pattern B-1 being the most frequent (49%; 53/108 strains), followed by subtype B-2 (34%; 37/108). Subtype B-1 was dominant during 2004 representing 76% (25/33) of studied isolates, then decreased during 2005 (24%; 7/29) and rose in 2006 to 48% (22/46). Subtype B-2 represented 52% (15/29) in 2005, and 48% (22/46) in 2006. No correlation could be established between rifampicin resistance levels and PFGE subtypes. This RIF-R clone was not restricted to a specific hospital ward. Isolates were obtained from patients admitted to intensive care, medical and surgical units. Almost all patients included in this study (101/108, 93%) acquired the MRSA in our hospital. Seven patients acquired the RIF-R MRSA infection or colonisation in a prior admission to another hospital.

### SCC*mec *typing, MLST and *spa *typing

SCC*mec *typing was carried out in the 32 strains where *rpoB *mutations were characterised. This selection included representatives of the eight PFGE B subtypes. Also RIF-S MRSA strains were analysed for SCC*mec *type. All 32 RIF-R MRSA strains carried a SCC*mec *type I. The 5 RIF-S of PFGE pattern A carried a SCC*mec *type IV-A. Interestingly, all strains belonged to a common MLST type: ST228, defined by alleles *arcc *1, *aroe *4, *glpf *1, *gmk *4, *pta *12, *tpi *24, and *yqi *29 (table 3).

**Table 3:** Molecular features and resistance patterns of multi-resistant MRSA isolates resistant and susceptible to rifampicin.

MLST (ST)	SCC*mec *type	PFGE	*spa*-type	Resistance pattern^1^
ST 228	I	B	t041	OXA, ERY, CLI, GEN, TOB, RIF, CIP
ST 228	IVA	A	t2222 or novel	OXA, ERY, CLI, GEN, TOB, CIP
ST 247	I	Iberian clone (ATCCBAA44; PER88)	t051	OXA, ERY, CLI, GEN, TOB, RIF, CIP, TET

(^1 ^OXA, oxacillin; ERY, erythromycin; CLI, clindamycin; GEN, gentamicin; TOB, tobramycin; CIP, ciprofloxacin; RIF, rifampicin)

In parallel, a selection of 18 RIF-R MRSA strains and the 5 RIF-S MRSA were further genotyped by *spa *typing. All RIF-R strains belonged to *spa*-type t041. Among the RIF-S MRSA strains, three belonged to *spa*-type t2222 and two showed novel *spa*-types (r26-r30-r17-r13-r17-r13-r17-r12-r17-r12 and r26-r30-r17-r20-r17-r12-r17-r12-r17-r16).

## Discussion

The multi-resistant nature of most MRSA clones found in hospitals represents a therapeutical challenge for treating serious MRSA infections. The burden that the Iberian clone posed in Spanish hospitals in the early 90 s [3,28], shifted to other clones susceptible to more antibiotics, which have been dominant in recent years [8,29]. In this paper, we described the emergence and spread of a MRSA clone resistant to clindamycin, erythromycin, gentamicin, tobramycin, ciprofloxacin and rifampicin which has reduced substantially the number of effective antibiotics for treatment of serious MRSA infections.

Rifampicin is an antibiotic of substantial interest in the rise of MRSA infections, but cannot be used as a single agent to treat such infections because rapid emergence of resistance can occur, even during therapy [16,30,31]. Although the role of rifampicin as adjunctive therapy is controversial [31], the combined therapy seems beneficial as long as the bacteria exhibit susceptibility to the antibiotics combined [30]. The distinctive phenotypic feature in the particular clone of ST-228 described here was the borderline resistance to rifampicin that could be missed by some methods of antimicrobial susceptibility testing (i.e. disk diffusion or E-test). Hence our interest in studying whether this low-level RIF-R was an adaptive phenomenon or to the contrary, known *rpoB *mutations underlay such phenotype.

Almost all isolates belonging to this multi-resistant MRSA clone (104/108) showed a low-level rifampicin resistance (MICs, 1 to 4 mg/L) and carried the amino acid substitution 481His/Asn in the RNA polymerase. Only 4 isolates showed additional substitutions known to be involved in a high-level rifampicin resistance: two isolates (MICs, 128 mg/L) carried mutational change 477Ala/Thr, and one isolate (MIC, ≥ 256 mg/L) 468Gln/Lys [13,17,27,32]. The fourth isolate (MIC, ≥ 256 mg/L) showed substitution 527Ile/Leu, the only one which mutation was found in the rifampicin resistance-determining cluster II, described recently among Japanese MRSA isolates [32]. It is noteworthy that 20 isolates (19%) of the RIF-R isolates, carrying r*poB *mutation resulting in amino acid substitution in position 481, were detected as rifampicin susceptible by the disk-diffusion test. However, the inhibition zones of these strains were between 20 and 23 mm, closer to the susceptibility breakpoint established by CLSI (susceptibility ≥ 20 mm) than inhibition zones among RIF-S MRSA isolates that were usually ≥ 30 mm. Therefore, if screening for rifampicin resistance is made only by disk diffusion, special attention needs to be paid to strains borderline to the CLSI susceptibility breakpoint to avoid reporting false susceptibility results. MICs by E-test failed to detect rifampicin resistance following CLSI guidelines [11] in a group of 12 strains (MICs, 0.75-1 mg/L). These isolates showed MICs by microdilution of 2 mg/L and carried the *rpoB *mutation responsible for amino acid substitution in position 481. Thus, and according to other authors, it would be advisable to apply ≤ 0.5 and ≥ 8 mg/L as new breakpoints to classify rifampicin susceptibility or resistance in *S. aureus *clinical isolates [13,17].

High-level rifampicin resistance could be attributable to double mutations within *rpoB*, as previously described [27]. We did not find in this particular clone that the presence of a prior mutational change (481His/Asn) increased the frequency of acquisition of additional mutations responsible for a higher level of rifampicin resistance, when compared to a reference strain.

The multi-resistance pattern exhibited by the Iberian clone, dominant lineage in our hospital during the 90's, also included resistance to tetracycline. The new RIF-R MRSA isolates were resistant to clindamycin, erythromycin, gentamicin, tobramycin, ciprofloxacin and susceptible to tetracycline. However, molecular typing showed that the Iberian clone and the new RIF-R MRSA clone had different genetic backgrounds represented by ST-247 and ST-228, respectively, with only a single locus in common. Although both clones carried a SCC*mec *element type I, PFGE patterns and *spa*-types were clearly different.

All strains with the multi-resistant phenotype described in this work, showing resistance or decreased susceptibility to rifampicin, belonged to ST-228, carried a SCC*mec *element type I and were *spa*-type t041. This clone seems to be related to the Southern Germany clone (ST-228, SCC*mec *type I, *spa*-type t001 or *spa*-type t041) reported in Germany in 1997-98 [21,33]. In the same period, strains of ST-228 and SCC*mec *type I were reported at several hospitals located in seven Italian cities [34], although these isolates also showed resistance to multiple antibiotics, rifampicin resistance was not stated. Recently, strains of ST-228 have spread epidemically in Finland in 2002-2004 and in Hungary in 2003-2004 [35,36]. Also, ST-228 has been reported in other European countries: Belgium, Slovenia or Switzerland [37]. The first isolate ST-228, SCC*mec *type I was isolated in our hospital in September 2003, from a patient admitted to the ICU. However, it was not until March 2004 that this clone spread epidemically in our hospital and currently represents one third of all clinical MRSA isolates in our institution. Strains belonging to ST-228 have been reported in other hospitals in Spain since 1996 [9,29,38]. However, none of these reports (from Spain or other countries) analysed the decreased susceptibility to rifampicin among representative strains of ST-228. During the 2004-2007 period, we did not find significant changes in the rifampin consumption in our institution, which was on average 0.5 DDD/100 patients-days for intravenous and 1.0 DDD/100 patients-days for oral administration.

A set of 5 strains resistant to clindamycin, erythromycin, gentamicin, tobramycin, ciprofloxacin, but fully susceptible to rifampicin with MICs of 0.012 mg/L were included in this study. On average, this RIF-S pattern represented 4% of all MRSA isolated between 2004 and 2006, however this resistance phenotype can be traced back to 1999 in our hospital. The RIF-S isolates were classified as ST-228, the same as the RIF-R MRSA. Isolates of ST-228 (MLST, *arcc *1, *aroe *4, *glpf *1, *gmk *4, *pta *12, *tpi *24, and *yqi *29) belong to the Clonal Complex 5, as well as isolates of ST-125 (MLST, *arcc *1, *aroe *4, *glpf *1, *gmk *4, *pta *12, *tpi *1, and *yqi *54) which was the dominant MRSA clone in Hospital Universitari de Bellvitge from 1996 to 2003. Consequently, an alternative hypothesis to explain the emergence of multiresistant clone ST-228, SCC*mec *type I would be the SCC*mec *type I transfer from ST-247 to the ST-125 background, with an intermediate stage where RIF-S isolates could be found belonging to ST-228.

## Conclusion

Our study describes the hospitalary spread of an MRSA clone (ST-228, SCC*mec*-I, *spa*-t041), related to the Southern-Germany clone (ST-228, SCC*mec *type I, *spa*-type t001 or *spa*-type t041) [21,33]. In this particular case, the studied strains were resistant to many more antibiotics than any previous MRSA clone spread in our institution, with the exception of the Iberian clone. In addition, the study of the *rpoB *mutations demonstrated that rifampin was not a suitable option for treatment of infections caused by this clone.

## Authors' contributions

MD and JL conceived the study and participated in its design. MD, FT, RM, MP and JL participated in field and clinical aspects of the study. VM and MD carried out the molecular genetic studies and sequence alignment. MD and VM wrote the manuscript which was co-ordinated by JL and critically reviewed by FT, RM and MP. All authors read and approved the final version of the manuscript.
